# A Predictable Approach of a Rare and Frequently Misdiagnosed Entity: Laryngeal Nerve Schwannoma

**DOI:** 10.3390/healthcare10010059

**Published:** 2021-12-29

**Authors:** Iulian Filipov, Lucian Chirila, Mihai Sandulescu, Corina Marilena Cristache

**Affiliations:** 1 Department of Maxillofacial Surgery, “Queen Maria” Military Emergency Hospital, 9 Pietii Str., 500007 Brasov, Romania; iulian_filipov@yahoo.com; 2 Department of Dental Techniques, “Carol Davila” University of Medicine and Pharmacy, 8, Eroilor Sanitari Blvd., 050474 Bucharest, Romania; 3 Department of Oral and Maxillofacial Surgery, “Carol Davila” University of Medicine and Pharmacy, 19 Plevnei Ave., 010221 Bucharest, Romania; 4 Department of Implant Prosthetic Therapy, “Carol Davila” University of Medicine and Pharmacy, 19 Plevnei Ave., 010221 Bucharest, Romania; mihai.sandulescu@umfcd.ro

**Keywords:** virtual reality, diagnosis, peripheral nerve tumor, patient communication, minimally invasive surgery

## Abstract

(1) Background: Schwannoma, a mesenchymal neoplasm derived from Schwann cells that line peripheral nerve sheaths, has a challenging diagnosis, due to the non-specific medical history and clinical examination. Nowadays, virtual reality (VR) is increasingly more used for enhancing diagnosis and for preoperative planning of surgical procedures. With VR, the surgeon can interact, before any surgery, with a virtual environment that is completely generated by a computer, offering them a real experience inside a virtual 3D model. (2) Methods and Results: The aim of the present paper was to present a case of surgically removal of a schwannoma, which originated from the fibers of the superior laryngeal nerve, in a predictable and minimally invasive fashion, upon using VR for diagnosis and surgical procedure planning. (3) Conclusions: The current clinical report attracted the attention of including schwannoma in the possible differential diagnosis of a swelling in the anterior cervical region, mainly when a nonspecific radiological appearance is noticed, even with the use of multiple imaging modalities. Virtual reality can increase the predictability and success rate of the surgical procedure, being in the meantime a good tool for communication with the patient.

## 1. Introduction

Schwannomas, which were first described by Verocay in 1910 as peripheral nerve tumors [1], and later on, in 1935, named neurilemoma by Stout [2], are mesenchymal neoplasm derived from Schwann cells that line peripheral nerve sheaths. These masses usually arise from the side of any nerve, except for the two cranial nerves: the n. olfactory (I) and n. optic (II), according to Batsakis [3], and are unilobular, insidious, painless, slow-growing tumors [4].

Head and neck schwannomas routinely involve cranial nerves V, VII, IX, X, XI, and XII; sympathetic chain [5]; and brachial or cervical plexus [6], and are about 25% to 45% of the entire described localizations [7]. Among other less frequent localizations are mediastinum [8] and retroperitoneum [9,10].The exact causes are still unknown [11]. Moreover, Kennedy et al. described traumatic injury post hemithyroidectomy as cause of a recurrent laryngeal nerve schwannoma [12].

Usually schwannoma is a benign tumor, occurring at any age, but malignant change in head and neck has an incidence varying between 8 and 13.9% [6,13].

Diagnosis of this peripheral nerve tumor is challenging, due to the non-specific medical history and clinical examination. 

The common differential diagnoses for a swelling in the anterior and lateral cervical region include carotid body tumor, carotid aneurysm, schwannoma, branchial cyst, thyroid cyst, laryngocoele, pulsatile secondary lymph nodal swelling, and lipoma [14].

Malignant epithelial and nonepithelial neoplasms such as squamous cell carcinoma [15], melanomas [16], adenoid cystic carcinoma, non-Hodgkin’s lymphoma [17], different thyroid malignancy [18], or various ganglion metastasis should also be considered as possible pathological entities [19].

Computed tomography (CT), magnetic resonance imaging (MRI), and fine needle aspiration (FNA) are mainly used to avoid misdiagnosis [20,21]. 

Nowadays, virtual reality (VR) is increasingly more used in diagnosis and preoperative planning of surgical procedures; some examples come from orthopedic surgery [22,23], but also several applications in maxillofacial surgery have been described [24,25,26].

With VR, the surgeon can interact, before any surgery, with a virtual environment that is completely generated by a computer, offering them a real experience inside virtual 3D model, and the patient can be aware of the type of surgery, the uneventful complications, and the treatment’s alternatives or intra/postoperative reconstructions [26].

The collaboration between medical professionals, engineers, and software developers has facilitated the implementation of VR in clinical practice. 

The aim of this paper was to present case of surgically removal of a schwannoma, which originated from the fibers of the superior laryngeal nerve, in a predictable and minimally invasive fashion, upon using virtual reality for diagnosis and surgical procedure planning.

## 2. Case Report

We report a case of a 67-year-old woman who presented to the Maxillofacial Outpatient Department of Military Hospital Brasov, Romania, complaining of a neck “lump” and feeling a slight discomfort at manual compression for the past 7 months. 

### 2.1. Diagnostic and Virtual Planning

Clinical examination revealed an elastic, tender, and mobile tumor, located on the left side of the neck. The rest of the head and neck examination was normal. During anamnesis, the patient had no complaints of odynophagia, dysphagia, or hoarseness. The patient was non-smoker and there was no family history of any genetic diseases or malignancy. The patient’s medical history included arterial hypertension and age-related cataracts, and her body mass index (BMI) was 34. 

Before the admission, the patient underwent diagnostic panendoscopic evaluation in another medical center. The findings of a fiberoptic laryngoscopy, bronchoscopy, and esophagoscopy revealed no abnormal masses.

**Figure 1:**
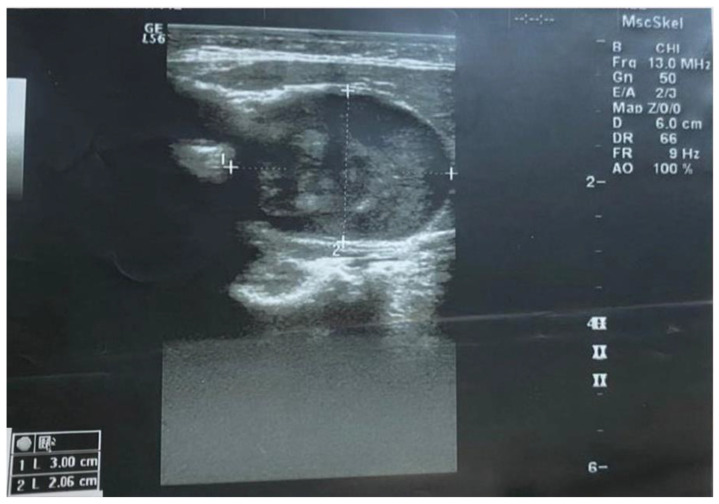
Ultrasound of the neck mass showing a thick-walled cyst with clear contents located on the left side, mesial to the sternocleidomastoid muscle. No flow was observed in the cyst or in the wall of the cyst upon ultrasound Doppler examination.

A neck ultrasonography revealed a 3 cm × 2.06 cm well-defined, hypoechoic mass (Figure 1), and no intratumoral vascularity was detected with Doppler imaging. 

**Figure 2:**
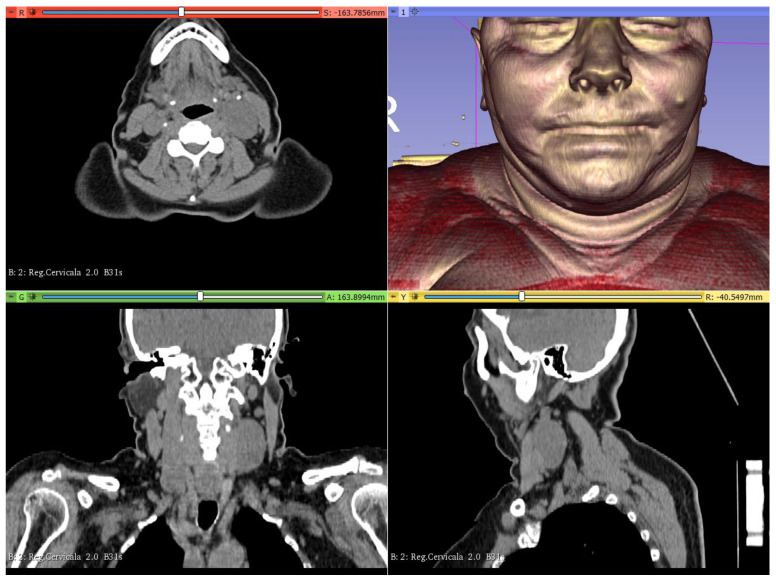
Axial view (red), coronal view (green), sagittal view (yellow), and 3D view (blue) of the head and neck computer tomography (CT) in 3D Slicer (www.3dslicer.org (accessed on 4 October 2021)) software. A well circumscribed cervical mass was observed in all sections. Asymmetric deformation of the left side of the neck was observed on the 3D view (blue).

A computer tomography (CT) scan of the head and neck region (Figure 2) unveiled a well-circumscribed cervical mass (4.5 cm × 3.2 cm × 2.7 cm), located under internal jugular vein (IJV), carotid artery (CA), and vagus (X) nerve. 

An ultrasonography-guided fine needle aspiration biopsy was performed in order to establish a pathologic diagnosis, but the result was non-diagnostic.

Thus, for avoiding misdiagnosis, Digital Imaging and Communication files (DICOM) from the CT scan were imported in an open-access multi-platform software, 3D Slicer (www.3dslicer.org (accessed on 4 October 2021)) [27], installed on a laptop computer with dedicated powerful graphic card (NVIDIA^®^ GeForce^®^ GTX 1650 Ti 4GB). From the extension manager, Slicer Virtual Reality was installed, enabling the user to interact with the 3D scene using virtual reality [28]. The extension was used with Oculus Rift headset (Meta Quest, Irvine, CA, USA), and a virtual navigation through registered anatomy, wearing VR glasses, was initiated to evaluate the limits of the left cervical mass.

**Figure 3:**
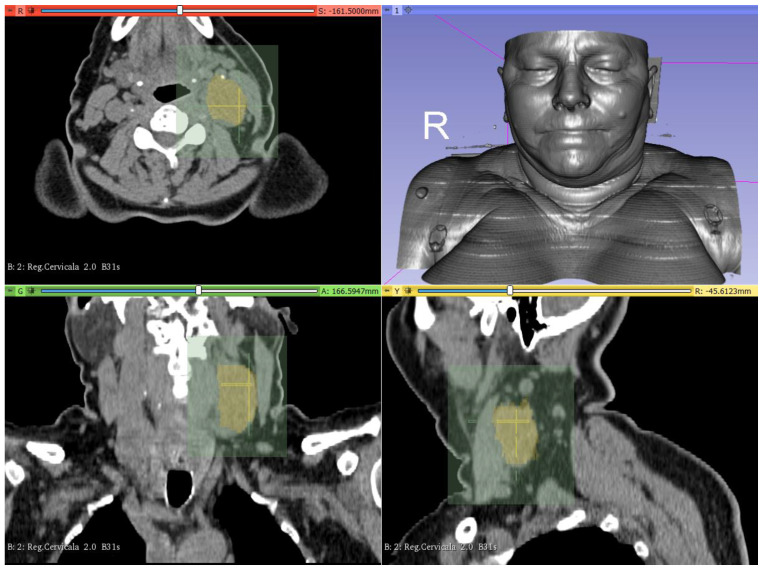
The abnormal cervical mass was segmented and could be clearly observed in axial (red), coronal (green), and sagittal (yellow) views. The Slicer Virtual Reality module was used to navigate inside the virtual 3D model for surgical treatment planning.

The Segment Editor module in 3D slicer software was used to manually segment the tumor for VR surgical removal (Figure 3).

All the information regarding the shape and position of the tumor as well as the closest vicinity with the superior laryngeal nerve, involving the risk of nerve resection with subsequent hoarseness, were presented to the patient. The informed decision was to perform surgical resection under general anesthesia, and the patient signed the consent form.

Before surgery, routine preoperative tests were performed according to National Institute for Health and Care Excellence (NICE) guidelines [29]. Blood test revealed high blood cholesterol levels (203 mg/dL), with LDL cholesterol level 120 mg/dL and fasting plasma blood glucose level (122 mg/dL). 

### 2.2. Surgical Procedures

**Figure 4:**
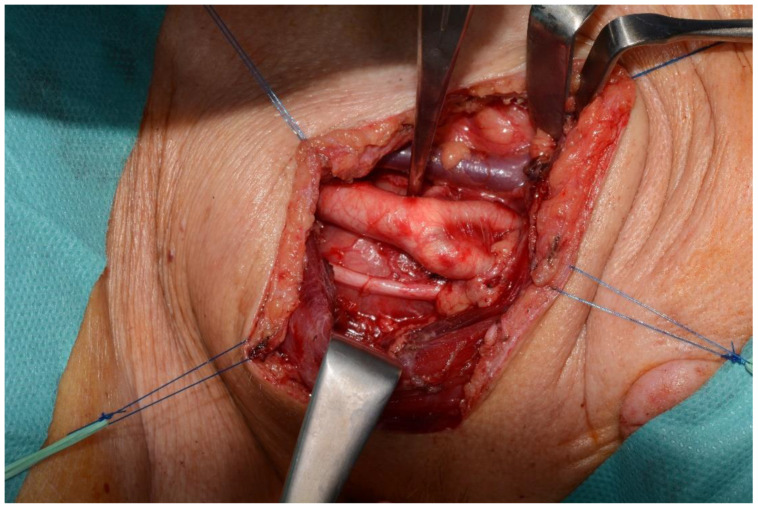
Lateral retraction of the sternocleidomastoid muscle. Underneath the vagus nerve, carotid artery (lateral), and internal jugular vein, the tumoral mass can be observed.

The anesthetic, 3 × 1.7 mL cartridges of Ubistesin™ Forte 1/100,000 (3M™ Espe, Saint Paul, MN, USA) solution, diluted with 4.9 mL of saline solution, was injected with a spinal needle (25-gauge × 3.5 inches) in a tumescent fashion, infiltrating the subcutaneous area. A 7 cm horizontal incision was made along a cervical skin crease into the lateral neck region. A subplatysmal sharp and blunt dissection was performed to dissect the over- lying soft tissue; the dissection was extended both cranially and caudally, taking care not to injure the internal jugular vein (IJV), carotid artery (CA), and vagus (X) nerve (Figure 4).

**Figure 5:**
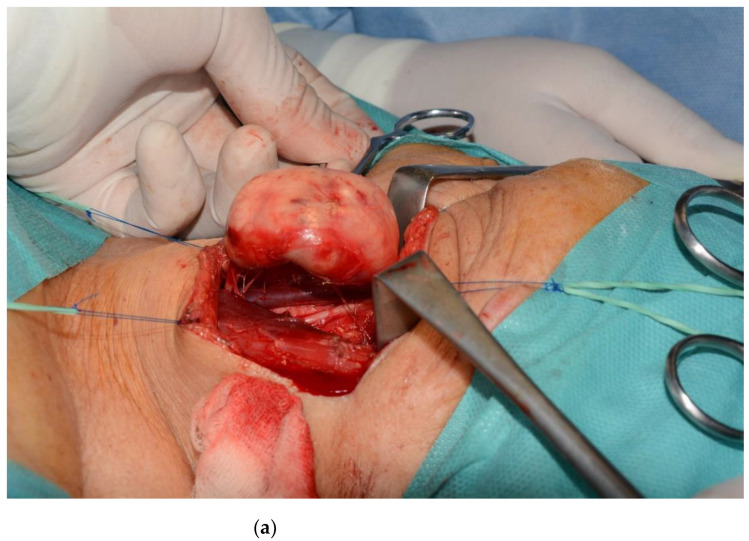

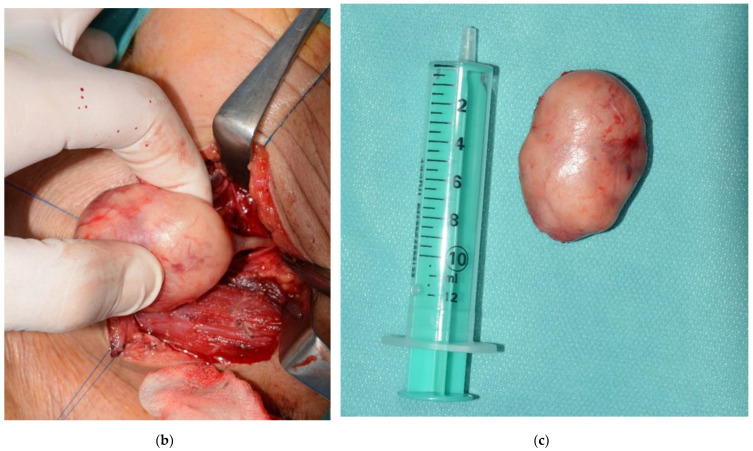
The well-incapsulated mass was micro surgically dissected from the superior laryngeal nerve (**a**). Several neural fascicles remain attached (**b**), and an “in-block” resection vas done; (**c**) the aspect of the tumoral mass upon its removal.

The carotid artery and the internal jugular vein were displaced laterally but not compressed. The sternocleidomastoid muscle was retracted laterally and a yellowish-white ovoid mass (4.5 cm × 3 cm) was identified; both the inferior and superior ends of the mass appeared in continuity with superior laryngeal nerve. A microsurgical approach was initiated in order to perform an intracapsular ablative procedure (Figure 5a,b). Since most of the neural fascicles were affected by the neoplasm, “in-bloc” resection was performed (Figure 5c).

The distance between the proximal and the cranial nerve stump of the superior laryngeal nerve was approximately 5.0 cm; direct coaptation could not be performed without significant tension and as such, a nerve cable graft was the only option to bridge the defect. Considering the fact that the patient did not consent to any course of action involving a nerve harvest procedure (neither from the same surgical field nor from a different donor site), the decision was not to perform any immediate nerve reconstructive procedure. However, both superior laryngeal nerve stumps were sutured to the lateral surface of sternocleidomastoid muscle, with a non-resorbable 5/0 suture (used as a mark for any secondary possible nerve reconstructive procedure, if it was the case).

### 2.3. Postoperative Care and Follow-Up

Postoperative recovery was uneventful. Due to minimally invasive procedure, the patient was discharged on the second postoperative day.

The pathological examination confirmed the diagnosis of benign schwannoma of the superior laryngeal nerve.

The patient developed a mild hoarseness following surgery and was diagnosed with vocal cord palsy by an ear, nose, and throat (ENT) specialist. She was informed, prior to surgery, both of the cause and the therapeutical options to treat the hoarseness. However, she expressed high satisfaction with the final outcome, without a personal significant decrease of her quality of life, and as such, did not request any further treatment. 

At the 12-month follow-up visit, there was no recurrent disease.

## 3. Discussion

Schwannomas, neurilemomas, neurolemmomas, or neurinomas are encapsulated nerve sheath tumors composed of proliferated Schwann cells, encompassing motor and sensory peripheral nerves. Their preoperative diagnosis is always a brain-teaser for both the surgeon and the radiologist.

Imaging examinations of schwannoma include ultrasound, CT, and magnetic resonant imaging (MRI). Recently, ^18^F-fludeoxyglucose (FDG) positron emission tomography (PET) was proposed, in association to MRI, for assessing tumor extension [30]. A precise and accurate preoperative diagnosis of neck schwannomas is unpredictable due to the nonspecific radiological appearance of these tumors, even with the use of multiple imaging modalities. Most of ultrasonography information described schwannomas as hypo echoic and well-defined lesions. Uniform enhancement is usual characteristic for schwannomas, whereas heterogeneous enhancement may be seen in larger lesions. Areas of heterogeneity may reflect cyst formation or necrosis. This raises the question of malignant degeneration, especially if the tumor is poorly demarked from the surrounding soft tissues [31,32].

Fine needle aspiration is a simple and safe procedure that can guide the clinician towards the optimal therapeutical approach. The major problem in this diagnostic procedure is to obtain enough and characteristic material for a good cytological examination [33]. However, this is not always the case, and thus the result can be interpreted as non-diagnostic.

Diagnosis certainty is achieved only with histological examination and immunohistochemical staining of a surgically resected specimen.

When different pathologies need to be taken into consideration, the evaluation of surrounding anatomical tissues is crucial. Three-dimensional evaluation of the surgical site, offered by the VR, improved the overall performance of the operation, particularly in terms of assessing the relations with vital neighboring structures.

Virtual reality is a popular technique, used nowadays in many areas of medicine and healthcare. 

Bartella et al. described the use, for the first time in the field of oral and maxillofacial surgery (OMFS), of VR glasses for evaluating three-dimensional imaging dataset (DICOM set taken from CT/cone-beamCT scans) [34]. The software used by the authors, MedicVR, developed by University of Applied Sciences, Aachen, Germany, was evaluated by a medical student, an OMFS resident, and an OMFS consultant. The usability of VR software and glasses to improve the preoperative understanding of three cases: a deeply impacted wisdom tooth, a fracture of the lower jaw, and an oncological resection, was rated. The general feedback was that the VR experience give the surgeon a good preoperative overview of the intraoperative findings [34]. 

The novelty in our case was the use of VR with an open and continuous developing software, 3D Slicer. In our case, VR navigation through registered anatomy and tumor segmentation was a very helpful tool for the evaluation of the extent of the tumor.

Virtual planning of surgery using VR and augmented reality is constantly developing [25]. Moreta-Martinez et al. proposed a step-by-step protocol enabling inexperienced users to create a smartphone app, which combines augmented reality and 3D printing for the visualization of anatomical 3D models of patients with 3D-printed reference markers for training, education, and surgical guidance [35].

The present case report highlighted the use of VR as aid for assisting in diagnosis of a swelling localized in the carotid triangle region. In spite of the clear limits of the tumoral mass with no intratumorally vascularity as diagnosis on Doppler imaging, the differential preoperative diagnosis in the absence of a cytological biopsy is impossible, due to several pathological entities located in this area. Thus, the choice for the present case was surgical ablation. 

The current report surveyed the clinical and imaging features of the tumor in order to spotlight possible diagnostic and management methods for this rare pathological entity. Due to the paucity of schwannomas, the degree of suspicion for this diagnosis is low. Not surprisingly, such tumors are subject to frequent misdiagnosis. Most of the scientific literature regarding laryngeal nerve schwannoma is represented by case reports [9] or case series with a limited number of patients [36]. Butleret al. retrospectively examined the clinical and pathologic features of head and neck schwannomas excised over a 20 year period at University of Michigan (Ann Arbor, MI, USA) and, among the 85 identified head and neck schwannoma, only a single case of laryngeal schwannoma was described [37]. This emphasizes the importance of incorporating schwannomas in the differential diagnosis when preoperative imaging studies reveal a neck tumor.

Cervical vagal schwannomas represent about 2–5% of neurogenic tumors [38]. According to Kshettry et al., the incidence is 2.93 per 100,000 in the 65–74-year-old age group in the USA [39].

Typically, a schwannoma comes from a single fascicle within the main nerve and dislocates outward from the rest of the nerve. Because the tumor arises within the nerve stealth, it is surrounded by a true capsule. Intracapsular dissection is recommended in order to keep the integrity of the affected nerve. For preventing recurrence, some authors suggested that the tumor capsule should be removed simultaneously [19]. Nevertheless, in cases with large schwannomas that invade most of the nerve fascicle, ”in-bloc” resection is the only possible treatment. Whenever possible, direct neuroplasty is indicated in order to avoid neurological complication. 

In our case, the ablative procedure was not possible without sacrificing the nerve. Hoarseness is the most common complication in our case of resection, as a result of injury of vagus nerve branches during surgery. The same approach was described by Synková et al., and the vocal cord palsy was treated by a phoniatrist, but with no expectation for reversal of the condition [9].

The 3D visual communication process between clinician and patient made possible with the VR was highly important. For the patient, it is crucial to understand the necessity of any additive surgical procedure (e.g., nerve reconstruction with or without cable graft) or the possible consequences for not doing it, and it is the clinician’s responsibility to inform the patient about possible diagnosis, treatment options, and complication risks. In the meantime, regarding the postoperative mild hoarseness installed, in our presented case report, the patient became used to it and did not accept the neuroplasty. However, vocal fold atrophy is one of the more common reported findings in elderly patients, with no tumoral pathology, also leading to dysphonia in older individuals [40].

Virtual reality represents a novelty in the oral and maxillofacial surgery and otolaryngology fields for diagnostic purpose and surgical planning; however, for extensive and predictable use, further investigations are needed.

## 4. Conclusions

The VR offered the users a real experience inside virtual 3D model, and it is a useful tool for diagnostic and comprehensive communication with the patient.

The current clinical report attracted the attention of including schwannoma in the possible differential diagnosis of a swelling in the anterior cervical region, mainly when a nonspecific radiological appearance is noticed, even with the use of multiple imaging modalities.
